# Prelamin A and ZMPSTE24 in premature and physiological aging

**DOI:** 10.1080/19491034.2023.2270345

**Published:** 2023-10-26

**Authors:** Howard J. Worman, Susan Michaelis

**Affiliations:** a Department of Medicine, Vagelos College of Physicians and Surgeons, Columbia University, New York, NY, USA; b Department of Pathology and Cell Biology, Vagelos College of Physicians and Surgeons, Columbia University, New York, NY, USA; c Department of Cell Biology, The Johns Hopkins University School of Medicine, Baltimore, MD, USA

**Keywords:** Aging, farnesyl, Hutchinson–Gilford progeria syndrome, lamin, mandibuloacral dysplasia, nuclear envelope, prelamin A, restrictive dermopathy, Ste24, ZMPSTE24

## Abstract

As human longevity increases, understanding the molecular mechanisms that drive aging becomes ever more critical to promote health and prevent age-related disorders. Premature aging disorders or progeroid syndromes can provide critical insights into aspects of physiological aging. A major cause of progeroid syndromes which result from mutations in the genes *LMNA* and *ZMPSTE24* is disruption of the final posttranslational processing step in the production of the nuclear scaffold protein lamin A. *LMNA* encodes the lamin A precursor, prelamin A and *ZMPSTE24* encodes the prelamin A processing enzyme, the zinc metalloprotease ZMPSTE24. Progeroid syndromes resulting from mutations in these genes include the clinically related disorders Hutchinson–Gilford progeria syndrome (HGPS), mandibuloacral dysplasia-type B, and restrictive dermopathy. These diseases have features that overlap with one another and with some aspects of physiological aging, including bone defects resembling osteoporosis and atherosclerosis (the latter primarily in HGPS). The progeroid syndromes have ignited keen interest in the relationship between defective prelamin A processing and its accumulation in normal physiological aging. In this review, we examine the hypothesis that diminished processing of prelamin A by ZMPSTE24 is a driver of physiological aging. We review features a new mouse (*Lmna*^L648R/L648R^) that produces solely unprocessed prelamin A and provides an ideal model for examining the effects of its accumulation during aging. We also discuss existing data on the accumulation of prelamin A or its variants in human physiological aging, which call out for further validation and more rigorous experimental approaches to determine if prelamin A contributes to normal aging.

## Introduction

Aging is an important determinant of osteoporosis and cardiovascular disease. A large percentage of the older U.S. population has at least one of these two disorders, making them enormous burdens in terms of suffering and medical costs [1–4]. A better understanding of the molecular drivers of bone and vascular decline during aging, through the analysis of relevant cellular and mouse models, will significantly hasten the development of strategies and therapeutics to improve public health of our older population. Research on progeroid syndromes has pointed to unexpected drivers promoting pathologies associated with these rare diseases and possibly with physiological aging more generally [5–7]. Progeroid syndromes caused by mutations in *LMNA*, which encodes prelamin A as well as lamin C, and *ZMPSTE24*, which encodes the prelamin A processing enzyme, the zinc metalloprotease ZMPSTE24, have implicated unprocessed, farnesylated prelamin A or its variants in accelerated aging processes [8,9]. However, much remains to be learned about the underlying pathogenic mechanisms these proteins mediate and their contributions, if any, to physiological aging.

## Defective processing of prelamin A and progeroid syndromes

**Figure 1.**
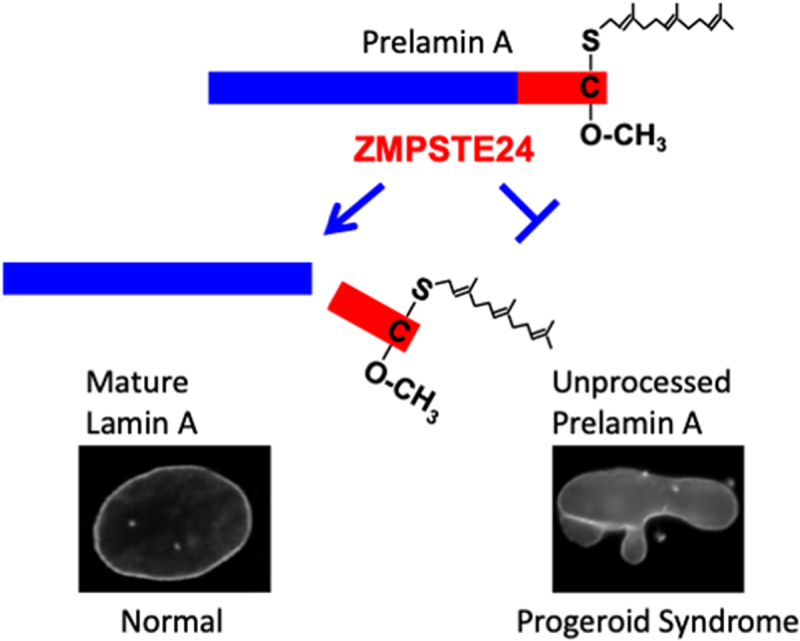
The processing of prelamin a in normal and progeroid cells. The post-translational processing steps leading to farnesylated and carboxyl methylated prelamin a (top) are described in the text. The final enzymatic reaction in prelamin a processing is the ZMPSTE24-catalyzed cleavage of prelamin A to generate mature lamin A (blue) and a 15-amino acid farnesylated and carboxyl methylated polypeptide (red) (left). This cleavage does not occur in cells lacking ZMPSTE24 or with a with a *LMNA* mutation that blocks cleavage (right). The farnesylated and carboxyl methylated cysteine is indicated. At bottom, fluorescence micrographs show that nuclei from cells expressing GFP-lamin a have a normal ovoid shape (left) whereas nuclei from cells expressing an unprocessed form of GFP-tagged prelamin a are aberrantly shaped (right).

Prelamin A, the precursor of the nuclear lamin A, has a carboxyl-terminal cysteine-aliphatic-aliphatic-any amino acid (CAAX) motif. The CAAX motif signals a series of three post-translational reactions. In the first step, protein farnesyltransferase catalyzes the addition of a farnesyl moiety to the cysteine [10,11]. Next, the RCE1 protease catalyzes cleavage between the farnesylated cysteine and -AAX residues, which are -SIM in prelamin A (it should be noted that ZMPSTE24 can also cleave some CAAX motifs, but not that of prelamin A) [12]. Third, the carboxyl-terminal farnesylcysteine is methylated in a reaction catalyzed by isoprenylcysteine carboxyl methyltransferase [13,14]. This series of reactions yields farnesylated and carboxyl methylated prelamin A (Figure 1, top). Most CAAX motif-containing proteins require these modifications for proper membrane binding or protein–protein interactions. However, prelamin A is unusual in that immediately after its synthesis a multispanning membrane enzyme, the zinc metalloprotease ZMPSTE24, catalyzes a second reaction that cleaves off the last 15 amino acids, producing non-farnesylated mature lamin A that incorporates into the nuclear lamina [9,15–18] (Figure 1, bottom left). The cleaved farnesylated and carboxyl methylated peptide has not been detected in cells and is likely rapidly degraded. The reason prelamin A undergoes modification followed by rapid cleavage of the modification is unknown, although it is clearly important for human health. When cleavage by ZMPSTE24 is blocked, farnesylated and carboxyl methylated prelamin A accumulates, leading to progeroid syndromes. At least one hallmark of progeroid cells is aberrantly shaped nuclei [19,20] (Figure 1, bottom right).

ZMPSTE24 and the integral membrane CAAX processing components are dually localized to both the inner nuclear membrane and endoplasmic reticulum membrane. Prelamin A contains a strong nuclear localization signal and its posttranslational modifications occur within the nucleus [21]. However, when prelamin A nuclear localization is artificially blocked by deletion of its nuclear localization signal, it nevertheless can be fully processed at the endoplasmic reticulum membrane [21,22].

## Prelamin A-related progeroid syndromes

**Figure 2.**
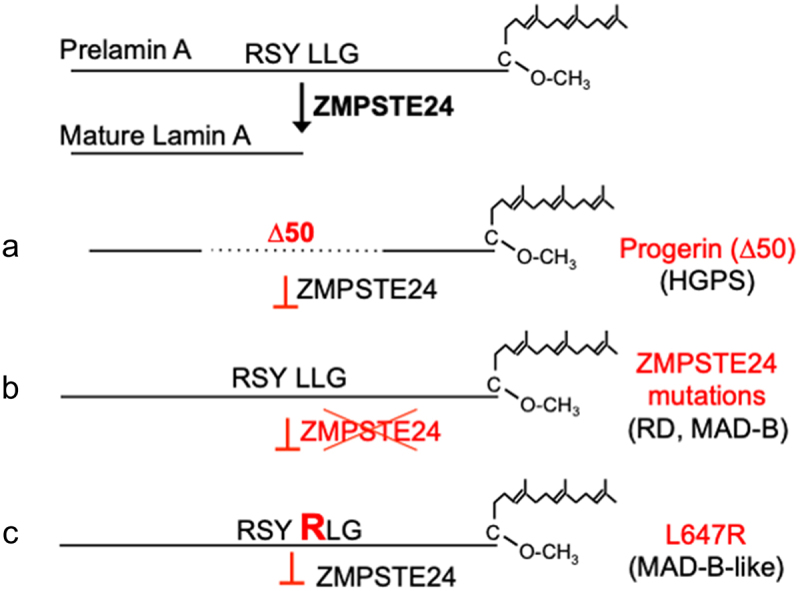
Defective prelamin a processing results in permanently farnesylated prelamin a and progeroid syndromes. Processing of human prelamin A to mature lamin A by ZMPSTE24, between residues Y_646_ and L_647_, is shown at the top. (a) a dominant *LMNA* splicing mutation internally deletes 50 amino acids, including the ZMPSTE24 processing site, resulting in progerin, and causing HGPS. (b) recessive mutations in *ZMPSTE24* leads to accumulation of unprocessed prelamin A, causing RD and MAD-B. (c) the dominant *LMNA*-L647R mutation disrupts ZMPSTE24-catalyzed cleavage, resulting in accumulation of prelamin A, and causing a MAD-B-like disorder.

Normally, farnesylated prelamin A is a transient species, essentially undetectable in cells because of its efficient conversion to mature lamin A. The premature aging disease Hutchinson–Gilford progeria syndrome (HGPS) is caused by a splicing mutation in *LMNA* that generates an internally deleted prelamin A variant called progerin or ∆50 prelamin A, but normal lamin C [23,24]. Progerin retains its CAAX motif, but lacks the ZMPSTE24 cleavage site, and thus remains permanently farnesylated and carboxyl methylated (Figure 2a). Children with HGPS manifest numerous premature aging symptoms, including generalized lipodystrophy, alopecia, failure to thrive, bone loss, and early-onset cardiovascular disease. Myocardial infarction or stroke is typically the cause of death for patients with HGPS and occurs in the mid-teens [6,25,26]. The progeroid syndromes restrictive dermopathy (RD) and mandibuloacral dysplasia-type B (MAD-B) result from mutations in *ZMPSTE24* that lead to accumulation of full-length permanently farnesylated and carboxyl methylated prelamin A (Figure 2b) [27–30]. RD, caused by complete loss-of-function mutations in *ZMPSTE24*, is neonatal lethal, presumably because of severe embryonic developmental defects in bone, skin, and other organs. MAD-B caused by partial loss-of-function mutations in *ZMPSTE24* is generally less severe than HGPS, with the lifespan of patients typically varying from the teens to the 40s. Distinct *ZMPSTE24* alleles retain varying levels of residual proteolytic activity that scale with disease severity [31,32]. MAD-B is characterized by growth retardation, partial lipodystrophy, mottled pigmentation of the skin, and prominent bone defects including mandibular hypoplasia and progressive resorption of bone of the distal phalanges and clavicles. A single patient has been reported whose symptoms are phenotypically similar to MAD-B but whose genetic mutation is in *LMNA* [33]. This patient with MAD-B-like disease has a heterozygous *LMNA* L647R point mutation that abolishes the ZMPSTE24 cleavage in human prelamin A, and thus also leads to accumulation of full-length farnesylated and carboxyl methylated prelamin A with a single amino acid substitution (Figure 2c). The patient had growth retardation, mandibular hypoplasia, mottled pigmentation of the skin, mild subcutaneous lipoatrophy, and resorption of a distal phalanx at 17 years of age. Echocardiography showed normal left ventricular ejection fraction and stress echocardiography showed no ischemic changes at 81% of the target heart rate, suggesting no coronary artery obstructive disease at that age.

## The farnesyl moiety promotes the toxicity of progerin and prelamin A

Considerable data implicate the farnesylated forms of prelamin A and its variants as dose-dependent drivers of progeroid phenotypes. It is thus the presence of permanently farnesylated forms of these proteins, and not the decrease in mature lamin A, that causes progeroid cellular defects and disease. Rather, loss of mature lamin A due to homozygous null *Lmna* mutations in mice or *LMNA* haploinsufficiency in humans causes cardiomyopathy and muscular dystrophy [34,35].

The first evidence for permanently farnesylated prelamin A being a driver of pathology came from studies of *Zmpste24*^−/−^ mice, which express prelamin A but no mature lamin A and provide a model for the human disease RD (but the mice survive past birth). Crossing these mice onto an *Lmna*^+/-^ background leads to accumulation of only half the amount of farnesylated prelamin A, and this is sufficient to eliminate essentially all progeroid symptoms [36,37]. Several groups then showed that treatment of cultured cells with protein farnesyltransferase inhibitors (FTIs) improves the abnormal nuclear morphology associated with accumulation of permanently farnesylated prelamin A or progerin [38–42]. In pioneering *in vivo* experiments, Fong et al. [43] demonstrated that an FTI improved progeroid phenotypes of *Zmpste24*^−/−^ mice. FTIs were later demonstrated to improve progeroid phenotypes in various mouse models of HGPS [44,45]. In MAD-B, the greater the residual activity of ZMPSTE24 due to different point mutations – resulting in more prelamin A processing – the lesser the severity of disease [31,32]. Clinical trials based on this preclinical research eventually led to the U.S. Food and Drug Administration approval of the FTI lonafarnib (brand name Zokinvy) for HGPS, MAD-B, and related progeroid syndromes with accumulation of prelamin A variants [46–48]. Preclinical data suggest that blocking carboxyl methylation of the isoprenyl cysteine in prelamin A may also be beneficial [49,50].

## How might permanently farnesylated prelamin A lead to progeroid phenotypes?

In order to understand how accumulation of permanently farnesylated prelamin A or its variants cause progeroid phenotypes, we must rely on additional basic research to decipher the functions of the nuclear lamina. The lamina is a meshwork of atypical intermediate filaments underlying the nuclear envelope inner membrane that in most mammalian differentiated cells is composed of mature lamin A, lamin B1, lamin B2, and lamin C [51,52]. Each of these lamins appears to form separate filaments, all of which contribute to the lamina. Lamin A is specifically implicated in numerous fundamental functions, including maintaining the structural integrity of the nucleus and providing an organizing platform for chromatin and transcription factors [53].

Lamin A also interacts directly with Sun proteins of the inner nuclear membrane [54]. In the perinuclear space, Sun proteins bind to outer nuclear membrane KASH (for Klarsicht, ANC-1, Syne homology) domain proteins, called nesprins in mammals, to form the ‘linker of the nucleoskeleton and cytoskeleton’ (LINC) complex [54–56]. Different nesprins bind to actin, microtubule motors, or intermediate filaments in the cytoplasm [57]. Hence, lamin A is part of an elaborate structural network connecting the inside of the nucleus to the cytoskeleton that functions in mechanotransduction and positioning of the nucleus within the cell [58,59]. Mechanical stress from outside the cell, such as the stiff extracellular matrix in bone and shear stress of blood flow in arteries, is therefore communicated to cytoskeletal elements and ultimately to lamin A through this network. This may lead to changes in genome organization and gene expression in response to extracellular mechanical stimuli [60]. Inputs from this mechanotransduction system may regulate the expression of lamin A itself, as its levels are increased in nuclei of cells in tissues of greater stiffness such as bone [61]. Farnesylated progerin prevents proper positioning of the nucleus in migrating fibroblasts, suggesting that it interferes with proper mechanotransduction between the extracellular matrix, cytoplasm, and nucleus [62].

The structural, transcriptional, or mechanical impacts of permanently farnesylated prelamin A or progerin that are directly responsible at a molecular level for the premature aging symptoms apparent in progeroid diseases have not been precisely determined. Alterations in numerous cellular processes and pathways have been reported in tissue culture cells or patient fibroblasts expressing permanently farnesylated prelamin A or progerin. Among the best documented of these are DNA replication stress, DNA repair defects, altered nuclear mechanics epigenetic alterations, telomere attrition, genomic instability, oxidative stress, stem cell exhaustion, cellular senescence, loss of proteostasis, increased inflammation, and mitochondrial dysfunction [63–79]. Notably, many of these defects and changes are strikingly similar to the classical ‘hallmarks of aging’ [80,81]. Thus, as discussed below, at least some of the changes that occur in physiological aging could result from diminished prelamin A processing by ZMPSTE24.

## Mouse models of prelamin A-related progeroid syndromes and their limitations

HGPS mouse models have been valuable in analyzing tissue-specific and systemic effects of progerin [44,45,82–89]. These HGPS mutant mice recapitulate most of the phenotypes characteristic of patients, including failure to thrive, alopecia, generalized lipodystrophy, bone defects, and vascular changes. The latter include smooth muscle cell loss and adventitial thickening in the aorta [45,82–89]. In contrast, endothelial cells, despite being the first to experience shear stress, remain preserved, albeit potentially functionally compromised [90–92]. Most strains of HGPS mice die prematurely between 4 and 10 months of age, with median survival varying between strains.

*Zmpste24*^−/−^ mice that accumulate farnesylated prelamin A have been used as a model for progeroid syndromes, sometimes interchangeably (and inaccurately) with HGPS [15,16,43]. *Zmpste24*^−/−^ mice fail to thrive, have severe bone defects, and die at 5–7 months of age. While prelamin A, like progerin, may cause vascular defects, this has not been established, as *Zmpste24*^−/−^ mice may die of other causes before this phenotype is manifest. A critical often overlooked problem in using *Zmpste24*^−/−^ mice is that the observed abnormal phenotypes may not be solely due to the accumulation of farnesylated prelamin A. ZMPSTE24 appears to have additional and critical cellular functions, one of which is clearing Sec61 translocons that are ‘clogged’ with partially folded nascent secretory proteins [93,94]. Loss of such a fundamental role in cellular quality control may confound the use of *Zmpste24*^−/−^ mice in understanding the specific role of prelamin A in pathology. ZMPSTE24 May also have other cellular roles; for instance, it functions through an as yet undefined mechanism to protect cells against enveloped viruses [95]. In the budding yeast *Saccharomyces cerevisiae*, where ZMPSTE24 was discovered and is called Ste24 [96–98], a *ste24* deletion mutation causes incorrect topology of a membrane reporter protein, induction of the unfolded protein response pathway, and improper secretion of proteins lacking a signal sequence [99–102].

## *Lmna*^L648R/L648R^ mice – a new model to study the impact of solely full-length farnesylated prelamin A

**Figure 3.**
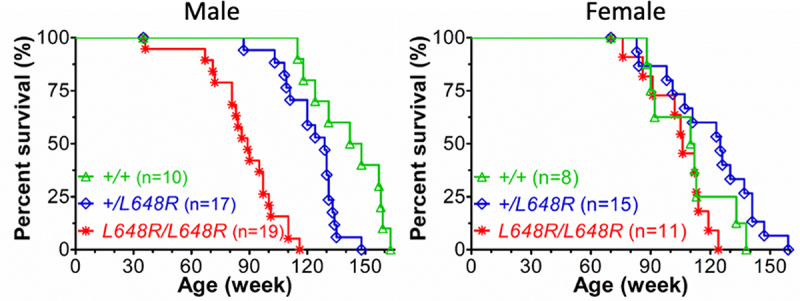
Survival curves for male and female *Lmna*^+/+^ (+/+), *Lmna*^+/L648R^ (+/L648R), and *Lmna*^L648R/648R^ (L648R/L648R) mice.Data are an extension of our originally published cohort [103].

We have overcome some of the limitations of the current mouse models to study prelamin A during aging by generating a new mouse model expressing an uncleavable prelamin A variant with a single amino acid substitution [103]. The mouse *Lmna*L648R allele is equivalent to the *LMNA*L647R allele in the previously characterized human patient with a MAD-B-like phenotype (Figure 2c) [33]. It changes the leucine just downstream from the processing site in the portion of prelamin A that is normally cleaved off and blocks the cleavage step. This leads to the expression of a permanently farnesylated prelamin variant with an arginine in place of a leucine at residue 648, and no mature lamin A. In humans, many *LMNA* mutations, including the classical HGPS mutation, are dominant and thus manifest disease in the heterozygous state. In mice, HGPS and other *LMNA*-based diseases are well modeled in the homozygous state, with heterozygotes of some strains showing more modest or later-onset phenotypes [44,83,86,104–106]. Thus, it is not surprising that *Lmna*^+/L648R^ mice are indistinguishable from wild-type littermates. In contrast, the homozygous *Lmna*^L648R/L648R^ mice develop failure to thrive and bone defects that are similar to, albeit less severe and of later onset, than those in *Zmpste24*^−/−^ mice. Strikingly, however, the *Lmna*^L648R/L648R^ mice have near-normal longevity, with male mice having a median survival of 89 and female mice 106 weeks (Figure 3). This finding is in stark contrast to *Zmpste24*^−/−^ mice that die at 20 to 28 weeks of age. The earlier death of *Zmpste24*^−/−^ mice may be due to loss of the other ZMPSTE24 functions discussed in addition to accumulation of farnesylated prelamin A. It is of note that the median survival of male *Lmna*^L648R/L648R^ mice is shorter than that of their female couterparts, which could result from a sex-specific difference in the biological effects of the L648R form of prelamin A or, more trivially, from more aggressive male mice attacking weaker cage mates. In any case, because *Lmna*^L648R/L648R^ mice grow old, they provide an ideal model for determining how prelamin A accelerates bone loss and possibly cardiovascular disease or other abnormalities that occur with aging.

**Figure 4.**
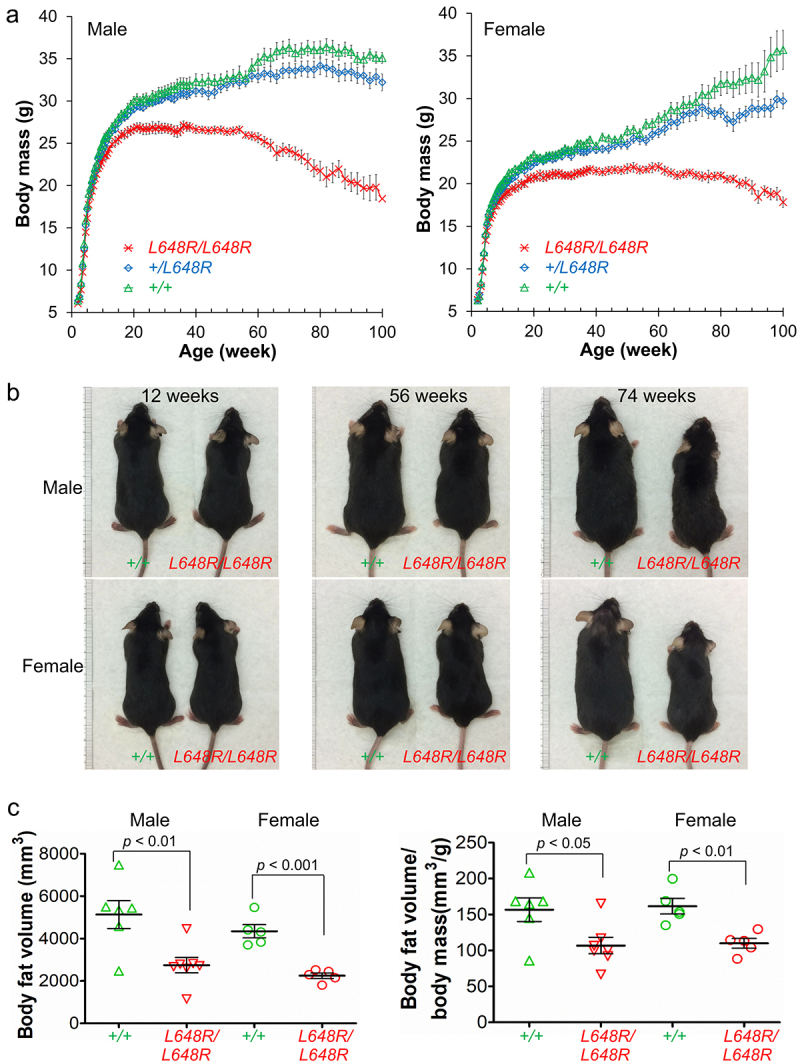
Failure to thrive in male and female *Lmna*^L648R/L648R^ mice. (a) plots of body mass versus age of male *Lmna*^L648R/L648R^ (L648R/L648R) (*n* = 20), Lmna+/L648R (+/L648R) (*n* = 17), and Lmna+/+ (+/+) (*n* = 13) mice and female L648R/L648R (*n* = 13), +/L648R (*n* = 15), and +/+ (*n* = 11) mice. Values are means and error bars indicate standard errors. (b) photographs of male and female +/+ and L648R/L648R mice at the indicated ages. (c) body fat volume (left) and body fat volume normalized to body mass (right) of 52-wk-old male and female +/+ and L648R/L648R mice. Triangles and circles represent values individual mice; the long horizontal bars represent means and error bars indicate standard errors. Figure is from Wang et al. [103].

*Lmna*^L648R/L648R^ mice show an overall failure to thrive. They have decreased body mass compared to *Lmna*^+/+^ mice starting at approximately 10 weeks of age that becomes more prominent over time (Figure 4a). Consistent with their decreased body masses, male and female *Lmna*^L648R/L648R^ mice are visibly smaller than *Lmna*^+/+^ animals (Figure 4b). They also have significantly reduced body fat at 52 weeks of age (Figure 4c).

**Figure 5.**
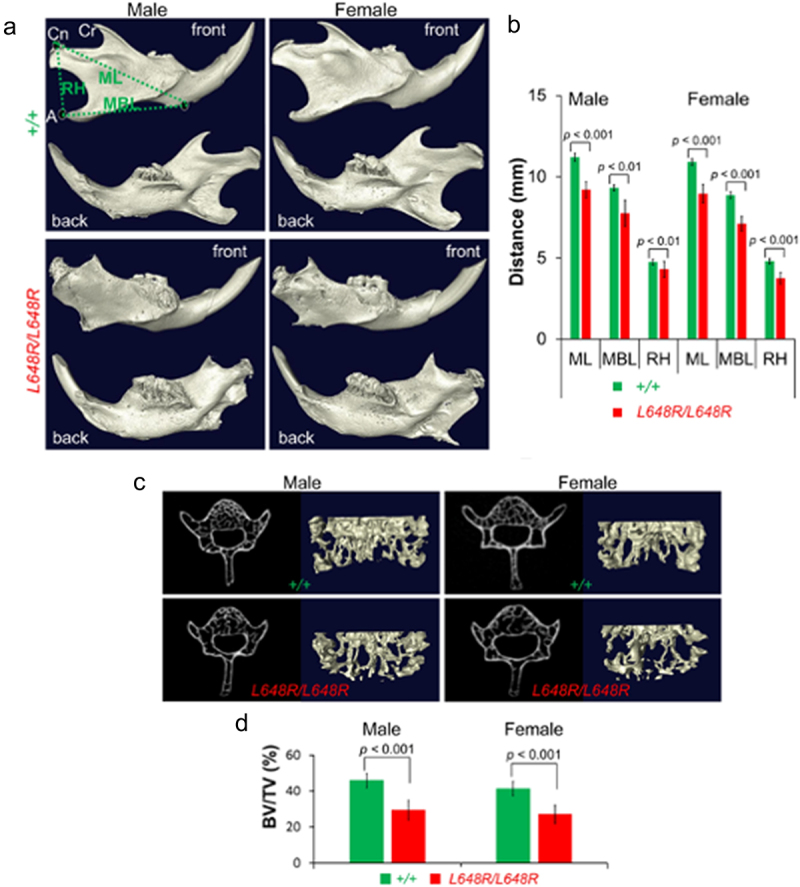
Bone defects in male and female *Lmna*^L648R/L648R^ mice. (a) Representative 3D renderings of the segmented micro-CT – scanned images showing mandibles of male and female *Lmna*^+/+^ (+/+) and *Lmna*^L648R/L648R^ (L648R/L648R) mice. A: angular process; cn: condylar process; cr: coronoid process; MBL: mandibular body length; ML: mandibular length; RH: ramus height. (b) comparisons of ML, MBL, and RH between male +/+ and L648R/L648R mice and between female +/+ and L648R/L648R mice. Values are means and error bars indicate standard errors. (c) Representative micro-CT – scanned transverse sections and 3D reconstructions of the L5 vertebrae from male and female +/+ and L648R/L648R mice. (d) comparison of vertebra L5 bone density (bone volume/total volume; BV/TV %) between male +/+ (*n* = 5) and L648R/L648R (*n* = 5) mice and between female +/+ (*n* = 7) and L648R/L648R (*n* = 6) mice. Values are means and error bars indicate standard errors. Figures are from Wang et al. [103].

The most prominent tissue-specific defects in *Lmna*^L648R/L648R^ mice are in bone, particularly in the cranium and mandible. The *Zmpste24*^−/−^ mice have what was described as ‘destroyed’ zygomatic arches at 8 weeks [15]. The more gradual onset of defects in the *Lmna*^L648R/L648R^ mice allowed us to demonstrate normal zygomatic arches at 4 weeks of age with degenerative changes secondary to bone resorption occurring at approximately 30 weeks of age [103]. Compared to *Lmna*^+/+^ mice, *Lmna*^L648R/L648R^ mice also demonstrate degeneration of the mandible and loss of vertebral bone density with age (Figure 5). These bone abnormalities in *Lmna*^L648R/L648R^ mice, which are significantly more severe than in age and sex matched *Lmna*^+/+^ mice, are reminiscent of temporomandibular joint degeneration and osteoporosis that occur in human physiological aging.

We do not know yet if *Lmna*^L648R/L648R^ mice develop vascular disease at old ages. As noted above, HGPS model mice have smooth muscle cell loss and adventitial thickening in the aorta [45,82–89]. While we have observed smooth muscle loss in two very old *Lmna*^L648R/L648R^ mice, the aortas of most of the mutant mice are histologically indistinguishable from those of their unaffected littermates. To observe vascular changes in *Lmna*^L648R/L648R^mice, it may be necessary to cross them to a mouse line susceptible to atherosclerosis, as has been done with one HGPS mouse model [88,107].

Until now, the biological effects of progerin and full-length prelamin A have been assumed to be similar, since both are permanently farnesylated, but the *Lmna*^L648R/L648R^ mice raise the possibility that there may be important differences between them. All of the prelamin A in *Lmna*^L648R/L648R^ mice is in the farnesylated and carboxyl methylated state (albeit with a single amino acid difference from wild-type prelamin A), yet their longevity is nearly normal, unlike HGPS mouse models or *Zmpste24*^−/−^ mice. However, the mechanism responsible for the longer lifespan is not known. With regard to differences with progerin, one hypothesis is that the loss of 50 amino acid may somehow render this permanently farnesylated variant more toxic than full-length, farnesylated prelamin A. Compared to wild-type prelamin A, the L648R amino acid substitution could potentially diminish the toxic effects of full-length, farnesylated prelamin A and its impact on longevity. While these and other hypotheses remain to be tested, this new mouse model nonetheless contrasts with existing HGPS and *Zmpste24*^−/−^ progeroid mice and facilitates the analysis of molecular and cellular changes caused by permanently farnesylated prelamin A. Given their near-normal longevity, *Lmna*^L648R/L648R^ mice also provide a powerful platform to study the effects of permanently farnesylated prelamin A with aging.

## Do prelamin A or progerin play a role in physiological aging?

The question of whether or not permanently farnesylated forms of prelamin A contribute to normal aging is an important one that remains as yet unsatisfactorily answered. Some studies have indeed claimed that prelamin A or progerin accumulate in cells of older humans. However, as discussed below, questions remain about the interpretation of those studies and none have been reproduced. This calls out for more rigorous and larger scale studies, particularly because existing therapeutic strategies for the premature aging diseases, *e.g*. FTIs, or new ones, could potentially be harnessed to improve health and increase lifespan in the normally aging population if a permanently farnesylated form of prelamin A were validated as driver of aging.

In 2006, Scaffidi and Misteli [64] showed that nuclei in skin fibroblasts obtained from old individuals acquired defects similar to those in cells from children with HGPS, including changes in histone modifications and increased DNA damage. Along with this, they detected progerin mRNA in fibroblasts from normal individuals, albeit at about 50-fold lower levels compared to those from patients with HGPS. However, progerin mRNA levels did not increase with age in normal fibroblasts, leaving open the question of whether it contributes to aging. Subsequently, McClintock et al. [108] reported that progerin accumulates in skin of aging individuals, and Olive et al. [109] presented data suggesting that it increases in coronary arteries in old age. However, the numbers of progerin expressing cells were extremely low in these studies, which have not been reproduced. Furthermore, other studies have found that the very low levels of progerin mRNA or protein in cells from individuals without HGPS do not increase with age [62,64].

Prelamin A has also been hypothesized to be involved in physiological aging. This is based on the compelling possibility that a slight decrease in ZMPSTE24 expression or activity could lead to prelamin A accumulation, which in turn could drive aging phenotypes such as osteoporosis. A frequently cited paper by Ragnauth et al. [110] concluded from an immunohistochemical comparison of arteries from young and old humans that prelamin A accumulates in the vasculature during physiological aging. These authors further provided immunohistochemical evidence that led them to conclude that smooth muscle cells in the media layer of vessels from young individuals contained high levels of ZMPSTE24 (and undetectable prelamin A), whereas arteries from aged individuals showed reduced ZMPSTE24 and an increased frequency of prelamin A-containing cells. However, this evidence is correlative, and furthermore the antibodies used in this study were not well validated. Lattanzi et al. [111] similarly reported that fibroblasts obtained from centenarians had decreased expression ZMPSTE24 and more of the fibroblasts in a culture were labeled with antibodies against prelamin A. While intriguing, the issue of rigor and reproducibility is critically important and studies such as these must be repeated with other protein detection approaches, including the use of different and well-validated antibodies for immunolabelling, and in larger numbers of subjects. Even if relatively low-level prelamin A expression is rigorously confirmed in cells of old individuals, it must be conclusively shown that the low levels present are detrimental, as heterozygous *Zmpste24*^+/−^ and *Lmna*^+/L648R^ mice appear to be asymptomatic. However, mice and humans differ in many ways, including overall lifespan and complications that occur with aging. It is also possible that aged human cells might be more sensitized to the presence of prelamin A and less able to neutralize its toxic effects, as has been proposed for progerin [64].

Studies of HIV protease inhibitors have also hinted at the possibility of a potential link between prelamin A accumulation and aging symptoms. Several HIV protease inhibitors, including lopinavir, inhibit ZMPSTE24, leading to the accumulation of permanently farnesylated prelamin A [112–117]. Patients infected with HIV on long-term antiretroviral therapy have an increased risk of atherosclerosis, myocardial infarction, and osteoporosis [118–120]. Prelamin A accumulation secondary to pharmacological treatment with these drugs could potentially therefore contribute to age-related complications, but a true causal link has not been demonstrated.

Studies of rare diseases and mouse models clearly implicate permanently farnesylated prelamin A in progeroid syndromes. If its connection to physiological aging is solidified, it raises speculation that FTI therapy could be a ‘fountain of youth’ that would prevent or retard age-related complications. Needless to say, the potential role of permanently farnesylated prelamin A in physiological aging warrants solidification with further rigorous and reproducible research.

## Data Availability

Data sharing is not applicable to this article as no new data were created or analyzed in this study.
